# A Review on Recent Trends in Green Synthesis of Gold Nanoparticles for Tuberculosis

**DOI:** 10.34172/apb.2021.002

**Published:** 2020-11-07

**Authors:** Arti Gupta, Sonia Pandey, Jitendra Singh Yadav

**Affiliations:** ^1^Uka Tarsadia University, Maliba Pharmacy College, Gopal Vidhya Nagar, Bardoli, Gujarat, India.; ^2^Shree Naranjibhai Lalbhai Patel College of Pharmacy, Umrakh, Gujarat, India.

**Keywords:** Green synthesis, Gold nanoparticles, Tuberculosis, Phytoconstituents

## Abstract

Tuberculosis (TB) is a contagious disease that has affected mankind. The anti-TB treatment has been used from ancient times to control symptoms of this disease but these medications produced some serious side effects. Herbal products have been successfully used for the treatment of TB. Gold is the most biocompatible metal among all available for biomedical purposes so Gold nanoparticles (GNPs) have sought attention as an attractive biosynthesized drug to be studied in recent years for bioscience research. GNPs are used as better catalysts and due to unique small size, physical resemblance to physiological molecules, biocompatibility and non-cytotoxicity extensively used for various applications including drug and gene delivery. Greenly synthesized GNPs have much more potential in different fields because phytoconstituents used in GNP synthesis itself act as reducing and capping agents and produced more stabilized GNPs. This review is devoted to a discussion on GNPs synthesis with herbs for TB. The main focus is on the role of the natural plant bio-molecules involved in the bioreduction of metal salts during the GNPs synthesis with phytoconstituents used as antitubercular agents.

## Introduction

**Figure 1:**
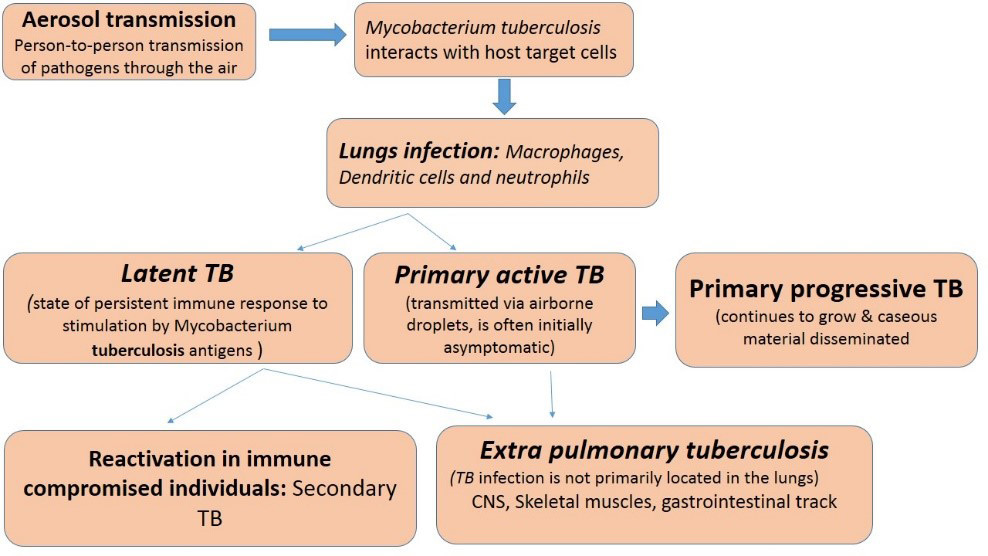
Tuberculosis infection and development.

Tuberculosis (TB) is a bacterial infectious disease caused by *Mycobacterium tuberculosis*, one of the oldest bacterial diseases. TB is still affecting and posing major health, social and economic burdens at the global level. However, low and middle-income countries are mainly affected. If the disease would not be managed efficiently then TB will be resurged due to some other diseases like HIV infection as well as multiple drug-resistant tuberculosis (MDR-TB) by considering these facts in 1993, the World Health Organization (WHO) took an unprecedented step and declared TB a global emergency.^1,2^ Synthetic anti-TB drugs are a two-edged sword while they destroy pathogenic *M. tuberculosis* they also select for drug-resistant bacteria against which those drugs are then ineffective1. TB either kills the infected individual or renders him/her incapable of assuming normal functions. Upon gaining entry into a new host, *M. tuberculosis* may result in an active infection or remain latent.^3^ TB is spread via various sources like infectious aerosols from an infected person. TB infections and their development are represented in Figure 1.

Wide ranges of phytoconstituents having the desired pharmacological effect on the body were responsible for anti-tubercular activity includes alkaloids^4-6^ glycosides^7-9^ glycoterpenoids,^10^ diterpenoids glycosides,^11^ tannins,^12^ phenolics and amides^13-18^ xanthones^19-23^ quinones,^24^ sterol^25-28^ triterpenoids.^29-37^ Terpenoids are scope for compounds that can be developed as future anti-mycobacterial drugs. It has been reported that ursolic and oleanolic acids are not so toxic and possess antimicrobial activity against some multi-resistant bacteria.^34,38-41^

Various antimycobacterial chemical compounds have also been isolated from plants, including ellagitannin punicalagin, allicin, and these compounds offered various clues for effective management of the disease to lessen the global burden of TB and drug-resistant *M. tuberculosis* strains.^42^ In this review, the author has emphasized the green synthesis of gold nanoparticles (GNPs) with herbs for TB (Antimicrobial and antibacterial activity). The main focus is on the role of the natural plant bio-molecules involved in the bioreduction of metal salts during the GNPs synthesis with phytoconstituents used as antitubercular agents. The plants having phytoconstituents acting as antitubercular agents discussed in Table 1.

**Table 1:** List of plants containing phytoconstituents having anti tubercular activity

**Botanical/family name**	**Phytoconstituents**	**References**
*Acalypha indica* (Euphorbiaceae)	Kaempferol, acalyphamide and other amides, quinone, sterols, cyanogenic glycoside	^43-47^
*Allium cepa* (Liliaceae)	Antibacterial substances (subterranean) allicin, ajoene indole alkaloids, steroidal triterpenes	^44,48-50^
*Allium sativum* (Liliaceae)	Sulphur containing amino acids known as alliin	^51,52-55^
*Adhatoda vasica (* Acanthaceae)	Vasicine acetate and 2-acetyl benzylamine, bromhexine and ambroxol, semi-synthetic derivatives of vasicine	^56,57^
*Aloe vera* (Liliaceae)	Anthraquinone glycosides (aloin),	^44,58^
*Berberis Hispanica* (*‎ Berberidaceae )*	-	^59^
*Byrsonima crassa* **(** Malpighiaceae)	Triterpenes:α-amyrin, β-amyrin and their acetates, lupeol, oleanolic acid,ursolic acid and α-amyrinone alkane dotriacontane, triterpenoids as bassic acid	^37,60^
*Buddleja saligna* (Scrophulariaceae)	Non-cytotoxic triterpenoids oleanolic	^61-63^
*Baccharis patagonica* (Asteraceae)	Oleanolic acid	^31^
*Clavijap rocera* (Theophrastaceae)	Oleanane triterpenoid (aegicerin)	^64^
*Canscora decussate* (Gentianaceae)	β-amyrin, friedelin, genianine, mangiferin, xanthones	^20,65^
*Colebrookea oppositifolia* (Lamiaceae)	dinor-cis-labdane diterpene and flavonoids	^66^
*Chuquiragau licina*	Lupeol	^31^
*Caesalpinia pulcherrima* (Rosaceae)	Furanoditerpenoids (6β-benzoyl-7β-hydroxyvouacapen-5α-ol, 6β-cinnamoyl-7β-hydroxyvouacapen-5α-ol) Flavonoid (myricitroside)	^67^
*Flacourtia ramontchii* (Flacourtiaceae)	Phenolic glucoside ester, (−)-flacourtin, ramontoside, β-sitosterol and its β- D-glucopyranoside	^1,65,68^
Junellia tridens (Verbenaceae)	Oleanonic acid	^31^
*Kalanchoe integra*, (Crassulaceae)	Triterpenoids- friedelin, taraxerol and glutinol and a mixture of long chain hydrocarbons Hypotensive, antiarrhythmic	^59^
*Leysera gnaphalodes* (Asteraceae)	Non-cytotoxic triterpenoids oleanolic	^62,39^
*Mallotus philippensis* (Euphorbiaceae)	Phloroglucinol derivatives; rottlerin, isorottlerin, isoallorottlerin	^68,69^
*Mimosa pudica*, (Mimosaceae)	Mimosine and turgorin	^68,70^
*Trichosanthes dioica* (Cucurbitaceae)	Amino acids, nicotinic acid, riboflavin, vitamin C, thiamine, 5-hydroxytryptamine	^70^
*Tinospora cordifolia* (Menispermaceae)	Alkaloids, carbohydrates, flavonoids, glycosides, lignin, saponins, terpenes, tannins, steroids	^71-74^
*Morinda citrifolia* (Rubiaceae)	Scopoletin, Anthraquinone salizarin and its glycosides, nordamnacanthol. Ursolic acid and β- sitosterol asperuloside and caproic acid	^75,76^
*Myrtus communis* (Myrtaceae)	Phenolic compounds	^77^
*Ocimum sanctum* (Labiatae)	Essential oil	^78-82^
*Prunus armeniaca* (Rosaceae)	Flavonoid glycosides, polyphenols, sterol derivatives, carotenoids, cynogenic glycosides and volatile compounds	^83,84,65^
*Piper species, Piper regnellii* (Piperaceae)	Piperine, neolignans, eupomatenoid-5, Aristolactams, dioxoaporphines, lignans, longamide, pluviatilol, methyl pluviatilol (fargesin), sesamin.	^85-87^
*Rumex hastatus* (Polygonaceae)	Naphthalene acylglucosides, rumexneposides.	^88^
*Salvia hypargeia* (Lamiaceae)	Diterpenoids (Labdane), hypargenin	^89-92^
*Senecio chionophilus* (Asteraceae)	Sesqui terpenoids (oxofuranoeremophilane)	^93,94^
*Vitex trifolia* (Verbenaceae)	Diterpenoids (halimane and labdane)	^1,95^
*Vitex negundo* (Verbenaceae)	Iridoid glycosides, isomeric flavanones and flavonoids	^96,97^
*Juniperus communis* (Cuppressaceae)	Isocupressic acid, communic acid and deoxypodophyllotoxin	^98,99^
**Monoterpenoids**		
Cymbopogon (lemon grass).	Citronellol, nero, dehydro costuslactone	^100^
**Sesquiterpenes**		
*Saussurea lappa* (Compositae)	Costunolide	^101^
*Magnolia grandiflora* (Magnoliaceae)	Parthenolide	^101^
*Ambrosia artemisiifolia* (Asteraceae)	11bH-dihydroparthenolide	^101^
*Ambrosia confertiflora* (‎Asteraceae)	Santamarine	^101^
*Sonchus hierrensis* (‎Asteraceae)	Santamarine	^101^
*Ambrosia confertiflora* (‎Asteraceae)	Reynosin	^101^
*Artemisia ramose* (Compositae)	Santonin	^101^
*Podachenium eminens* (Asteraceae)	7-hydroxydehydrocostuslactone	^102^
Zaluzania triloba (Compositae])	Zaluzanin C	^101^
**Diterpenes**		
*Tetradenia riparia* (Lamiaceae)	Sandaracopimara-8(14)-15-diene-7a,18-dio	^103^
*Juniperus excels* (Cupressaceae)	Sandracopimaric acid, juniperexcelsic acid	^104^
*Salvia multicaulis* (‎Lamiaceae)	12-demethylmulticauline, multicaulin, 12-demethylmultiorthoquinone, multiorthoquinone, 12-methyl-5-dehydrohorminone, 2-methyl-5-dehydroacetylhorminone, salvipimarone	^90^
*Azorella madreporica* (Apiaceae)	9,12-cyclomulin-13-ol	^105^
**Triterpenes**		
*Ajuga remot* a (Lamiaceae)	Ergosterol-5,8-endoperoxide	^106^
*Melia volkensii* (Meliaceae)	6b-hydroxykulactone, kulonate	^106^
*Borrichia frutescens* (Asteraceae)	(24R)-24,25-epoxycycloartan-3-one, (3b,24R)-24,25-epoxycycloartan-3-ol, (3b,24R)-24,25-epoxycycloartan-3-ol acetate, (23R)-3-oxolanosta-8,24-dien-23-o	^107^
*Sarmienta scandens* (Gesneriaceae)	Zeorin, 7b-acetyl-22-hydroxyhopane, 7b,22-dihydroxyhopane,	^31^
*Baccharis patagonica* (Asteraceae)	Oleanolic acid, erythodio	^31^
*Junellia tridens* (Verbenaceae)	3-epioleanolic acid, oleanonic acid	^108^
*Chuquiraga ulicina (* Asteraceae)	lupeol acetate, lupenone, 3-hydroxynorlupen-2-one, 3-acetoxynorlupen-2-one	^31^
*Acaena pinnatifida* (Rosaceae)	Pomolic acid, pomolic acid acetate, tormentic acid, 2-epi-tormentic acid, euscaphic acid, niga-ichigoside F1 aglycone	^31^

To avoid the adverse effect of recently used synthetic anti-TB drug^109^ natural products including plants, animals, and minerals have been the basis of treatment of human diseases 1. Studies showed that males with above 35 years of age of the patients, female, HIV-infected, older, and Asian-born patients are more prone to the major adverse effect of recent anti-TB drugs.^110^

Owing to the diversity of different natural active components such as plants, marine algae and types of metal salts and their ability to alter the composition of a reaction mixture through exposure to changes in the temperature, pH, and presence of various additives of biological origin (bio-matrices) which allows to produce nanoparticles of various metals with a defined size and shape.^111^ It is well established that biologically synthesized metal nanoparticles had various proved, biomedical applications like targeted delivery of cancer drugs, molecular imaging, wastewater treatment, cosmetics, as antiseptics, bio-sensors, antimicrobials, catalysts, optical fibers, agricultural, bio-labeling and in other areas is proved to be much safer, environment-friendly and cost-effective method of synthesis.^111-113^ Due to the diverse applications of Nanoparticles, several green approaches have been explored for synthesizing nanoparticles using different natural sources such as plants, marine algae, all these having immense tolerance to metal salts and have good ability to secrete extracellular enzymes for reduction of metals to consecutive nanoparticles.^113-115^ Gold is the most biocompatible metal nanoparticles are used in therapeutics and diagnostics in recent days to be studied in the recent field of bioscience.^115-119^ The biosynthesized GNPs were found to be better catalysts without using synthetic surfactant or capping agent at a low and definite concentration^120^ GNPs provide non-toxic carriers for drug and gene delivery applications. With these systems, the gold core imparts stability to the assembly, while the monolayer allows tuning of surface properties such as charge and hydrophobicity. An additional attractive feature of GNPs is their interaction with thiols, providing an effective and selective means of controlled intracellular release.^121^

**Figure 2:**
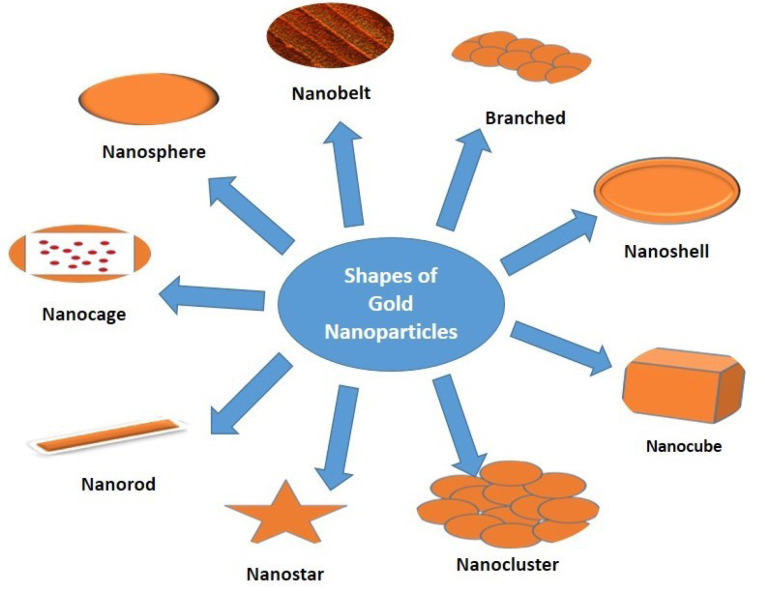
Different shapes of gold nanoparticles.

By controlling shape like nanospheres, nanorods, nanoshells, nanocages and structure of GNPs the surface plasmon resonance peaks of gold nanostructures can be tuned from the visible to the near-infrared region (solid vs. hollow). A combination of this optical tunability with the inertness of gold makes gold nanostructures well suited for various biomedical applications.^122^ The principle application of GNPs in the biomedical field is sensors,^123-125^ antimicrobials,^126-128^ catalysts,^129-131^ electronics,^132,133^ optical fibers,^134,135^ agricultural,^136-138^ bio-labelling^139^ development of specific scaffolds, conjugates to biomedical diagnostics and analytics, photothermal and photodynamic therapies, and delivery of target molecules.^140-142^ Different shapes (nanosphere, nanobelt, branched, nanocage, nanoshell, nanocubes, nanorod, nanostar, and nanocluster) of GNPs are represented in Figure 2 and their applications are discussed in Table 2.

**Table 2:** Shapes of gold nanoparticles and their applications

**Shape**	**Size**	**Applications**
Nano rod	2-5 nm	Photothermal Tumor Therapy, gas sensors^139,143^
Nano sphere	10-200 nm	(i) The development of an ultrasensitive nanoscale optical biosensor based on LSPR wavelength-shift spectroscopy and (ii) The SERS detection of an anthrax biomarker ^144^ Nanospheres used as targeted drug delivery on tumor and brain^144,145^
Nano star	46-76 nm	Inkjet printing technology,^146^ SERS sensor for Hg^2+^ detection^147^
Nano clusters	∼1.4 nm	Potential for cancer therapy,^148^ biological labelling applications^149^
Nano cube	50 nm	Biomedical Applications^150^
Branched particle	90 nm	Nanostars have been predicted and demonstrated to shine brighter than any other shapes, thus opening new avenues for highly sensitive detection or biolabelling, among other applications.^151^
Nanocage	36 nm nanocage	Photothermal cancer treatment, applications in nanobioelectronics,^152^ Biomedical Applications.^150^
Nanobelt	Thickness: 80 nm With: 20 nm Lenth: 0.15 nm	One-dimensional nano-scale sensors, transducers, and resonators.^153^
Nanoshell	≥100 nm	Fluorescent diagnostic labels, catalysis, avoiding photo degradation, enhancing photoluminescence, creating photonic crystals, preparation of bio conjugates, chemical and colloidal stability.^154^

## Green synthesis of gold nanoparticle

**Figure 3:**
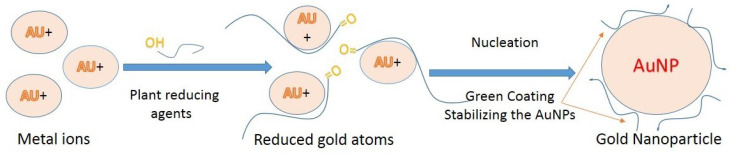
Green gold nanoparticles synthesis using plant/plants extract.

In the late 1990s, the development of non-toxic methods has embraced the principles of green chemistry.^155^ Green synthesis of metal nanoparticles has received widespread attention in the past decade due to its ability to meet environmental and economic goals simultaneously without using the chemical and cost-effective too. Green synthesis common approaches for GNPs have been shown in Figure 3. For the green synthesis of GNPs, the antioxidant components of the studied plant extracts are responsible for the reduction of metal salts, leading to the growth and stabilization of the GNPs.^156^

Medicinal herbs having phytochemicals like as alcohols, phenols, proteins, terpenes, alkaloids, saponins, etc which can act as reducing as well as capping agents in the GNPs biosynthesis.^157,158^

### Role of natural constituents for the green synthesis of GNPs

The triterpenes skeletons like cucurbitanes, cycloartanes, dammaranes, euphanes, friedelanes, holostanes, hopanes, isomalabaricanes, lanostanes, lupanes, oleananes, protostanes, tirucallanes, and ursanes^159^ are of interest ranging from primarily structural (cholesterol in cell membranes) to functional (carotenoids in photosynthesis, retinal in vision, quinones in electron transfer).^160^ Terpenoids play a crucial role in the reduction process of metal ions into nanoparticles, like eugenol the main terpenoid present in many plants.^111^

GNPs of *Schinus molle* L extract contain phenol, which shows that the differences in transmittance. Purified phenolics like gallic and protocatechuic acid act as reducing and capping agents in GNP synthesis because of the involvement of functional groups present in this phenolic compounds.^161-163^ These findings can help to determine the mechanism of metal nanoparticles by using crude extracts formation and stabilization. *Cinnamomum verum* extract contains polyols like (flavones and terpenoids) and polysaccharides, both contents act as reducing agent in metal ion synthesis.^164^ Flavonoids belong to the group of polyphenolic compounds that comprise several subgroups: anthocyanins, isoflavonoids, flavonols, chalcones, flavones, and flavanones, which can actively participate in the reduction and chelation of metal ions into nanoparticles. Literature established that reactive hydrogen atom release from tautomeric transformations of flavonoids from the enol-form to the keto-form can reduce metal ions to form nanoparticles. For example, flavonoids luteolin and rosmarinic acid present in *Ocimum basilicum* extracts it is the transform from the enol- to the keto-form.^111^ Apiin glycoside obtained from *Lawsonia inermis* used for the synthesis of anisotropic gold and quasi-spherical silver nanoparticle.^165^ The oxygen atoms belonging to 3-hydroxy and 4-oxo, and the 5-hydroxy and 4-oxo groups, are the preferred potential sites for chelation on quercetin.^166^

Many proteins contain active sites for metal ion accumulation and reduction where GNPs can form and be stabilized. In the process of nanoparticles formation, Protein donates electrons to react with metal ions and their subsequent stabilization that leads to the formation of nanoparticles.^167^ Some low molecular weight protein bands present in the soya bean extract, this may have been used up in biosynthesis of GNPs.^168^

The compounds present in the extracts can act as reducing as well as stabilizing agents and render more biocompatibility to the green synthesis of GNPs.^169^ High cost, use of environmentally hazardous chemicals, non-availability and presence of toxic capping agents limit the use of various physical and chemical methods.^170-171^ These limitations contributed the need for the development of new methods and materials for the production of nanoparticles based on the principles of ‘‘Green synthesis’’. The emphasis in this approach is on the synthesis and application of the nanoparticles for a maximum societal benefit, with minimal impact on the ecosystem.^172^

In Table 3 some part of plants which have been exploited by researchers for making AuNPs from the last decades have been summarised.

**Table 3:** List of synthesized gold nanoparticles using whole, parts or extracts of different plants

**Extract of plants**	**Part/ bomolecule**	**Size and shape**	**References**
*Allium cepa* L.	Vitamin C	~100 nm	^173^
*Anacardium occidentale* L.	Polyols and proteins	Hexagonal	^174^
*Azadirachta indica*	Nimbin, Azadirone, Azadirachtins	2-100 nm	^175^
*Camellia sinensis*	Polyphenolic compounds	25 nm	^176^
*Chenopodium album*	Oxalic acid	12 nm,10 nm	^177^
*Justicia gendarussa*	Polyphenol and flavonoid	27 nm	^178^
*Macrotyloma uniflorum* (Lam)	Aqueous extract	14-17 nm	^179^
*Mentha piperita* L	Menthol	90 nm, 150 nm	^180^
*Mirabilis* jalapa L.	Polyols	100 nm	^181^
*Swietenia mahogany*	Polyhydroxy limonoids	-	^182^
*Sapindus mukorossi*	Fruit pericarp	9 nm-19 nm	^183^
*Prunus domestica*	Fruit extract	14 nm-26 nm	^184^
*Magnolia kobus*	Leaf extract	5 nm-300 nm	^185^
*Coleus amboinicus lour*	Leaf extract	9.05 nm-31.95 nm	^186^
*Cassia auriculata*	Leaf extract	15 nm-25 nm	^187^
*Abelmoschus esculentus*	Seed, aqueous extract	45 nm-75 nm	^188^
*Zingiber officinale*	Rhizome extract	5 nm-15 nm	^189^
*Rosa hybrid Petal*	Petal extract	Petal 10 nm	^190^
*Cicer arietinum*	Been	Gold prisms (∼25 nm thick)	^191^
*Sugar beet*	Pulp	Nanowire	^192^
*Nyctanthes arbortristis*	Flower	19.8 ± 5.0 nm	^193^
*Gnidia glauca*	Flower	50 nm-150 nm	^170^
*Mangifera indica*	Peel extract	6.03-18 nm; spherical	^136^
*Gymnocladus assamicus*	pod extract	4-22 nm; hexagonal, pentagonal and triangular	^194^
*Cacumen platycladi*	---	Variable	^195^
*Coriandrum sativum*	Leaf	6.75-57.91 nm; spherical	^196^
*Nerium oleander*	Leaf extract	2-10 nm; spherical	^197^
*Butea monosperma*	-	10-100 nm; spherical, triangular	^198^
*Pea nut*	--	110 to 130 nm; variable	^199^
*Hibiscus cannabinus*	Stem extract	10-13 nm; spherical	^200^
*Sesbania grandiflora*	Leaf extract	7-34 nm; spherical	^201^
*Salix alba*	Leaf extract	50-80 nm	^202^
*Eucommia ulmoides*	Bark	Spherical	^203^
*Galaxaura elongata*	Powder or extract	3.85-77.13 nm; spherical, triangular, and hexagonal	^204^
*Ocimum sanctum*	Leaf extract	30 nm; hexagonal	^205^
*Torreya nucifera*	---	10-125 nm; spherical	^206^
*Olea europaea*	Leaf extracts	50-100 nm; triangular, hexagonal	^207^
*Rosa indica*	Rose petals	3-15 nm; spherical	^208^
*Pistacia integerrima*	Galls extract	20-200 nm	^209^
*Terminalia arjuna*	Fruit	60 nm, spherical	^118^
*Euphorbia hirta*	Leaf extract	6-71 nm, spherical	^210^
*Morinda citrifolia*	Root extract	12.17-38.26 nm, spherical	^211^
*Zizyphus mauritiana*	Extract	20-40 nm, spherical	^212^

### Role of microorganisms for the green synthesis of GNPs 

A variety of microorganisms are interacted with inorganic metals like gold, zinc, and silver and are known to use in bioleaching of minerals.^213^ Microbial cells treated with gold nanostructures synthesize by gold salts which are then isolated and purified using various techniques to obtain GNPs. Table 4 reflects a variety of microbes along with their genus which was used to make GNPs of different size ranges.

**Table 4:** List of microorganisms which have been used for synthesis of GNPs

**Microorganism**	**Genus**	**Size**	**References**
*Pseudomonas fluorescens*	*Bacterium*	50 nm–70 nm	^214^
*Shewanella algae*	*Bacterium*	10 nm–20 nm	^215^
*Geobacillus stearothermophilus*	*Bacterium*	-	^216^
*Escherichia coli DH5 α*	*Bacterium*	-	^217^
*Marinobacter Pelagius*	*Bacterium*	10 nm	^218^
*Stenotrophomonas maltophilia*	*Bacterium*	40 nm	^219^
*Rhodopseudomonas capsulate*	*Bacterium*	10 nm–20 nm	^220^
*Micrococcus luteus*	*Bacterium*	-	^221^
*Yarrowia lipolytica*	*Marine Yeast*	-	^222^
*Acanthella elongate*	*Sponge*	7 nm–20 nm	^223^
*Stoechospermum marginatum*	*Algae*	18.7 nm–93.7 nm	^224^
*Sargassum wightii Greville*	*Algae*	8 nm–12 nm	^225^
*Streptomyces viridogens*	*Bacterium*	18 nm–20 nm	^226^
*Candida albicans*	*Fungi*	20 nm–80 nm	^227^
*Aspergillus fischeri*	*Fungi*	50 nm spherical shaped	^112^
*Acanthophora spicifera*	Algae	-	^228^
*Chlorella pyrenoidusa*	Algae	-	^229^
*Kappaphycus alvarezii*	Algae	-	^230^
*Galaxaura elongata*	Marine alga		^203^
*Tetraselmis kochinensis*	Algae	5–35 nm	^231^
*Sargassum myriocystum*	Algae	15 nm	^232^
*Stoechospermum marginatum*	Algae	-	^223^
*Laminaria japonica*	Aqueous of extract Brown algae	-	^233^

### Role of biomolecules for the green synthesis of GNPs 

Biomolecules produced by living organisms to catalyze biological functions, such as nucleic acids, amino acids, lipids, and carbohydrates, possess hydroxyl and carbonyl functional groups in their structure which can reduce Au3+ ions to Au0 neutral atoms. These Au0 neutral atoms are then capped to form stabilized GNPs.^234^ This method can use for the biosafety of the reactants in GNPs synthesis. In Table 5 various biomolecules with type and size have been discussed.

**Table 5:** List of various biomolecules involved in synthesis of AuNPs

**Biomolecule**	**Type**	**Size (diameter)**	**References**
Linoleic acid	Fatty acid	10 nm	^235^
Tannic acid	Fatty acid	8 nm–12 nm	^178^
NADPH-dependent enzyme	Enzyme	25 nm	^236^
Aminodextran	Polysaccharide	18 nm–14 nm	^237^
Chitosan	Polysaccharide	-	^238^
Glucose	Carbohydrate	22 nm–38 nm	^239^
Sucrose, Raffinose	Carbohydrate	4 nm–16 nm, 30 nm–48 nm	^238^
Dextrose-encapsulated	Carbohydrate	25 nm, 60 nm, 120 nm	^240^
Starch	Polysaccharide	11 nm–15 nm	^241^
Bovine serum albumin	Protein	-	^242^
Serrapeptase	Protein	20 nm -200 nm	^243^
Trypsin	Enzyme	-	^244^
Glycosaminoglycans	Mucopolysaccharides	-	^245^
Serratiopeptidase	Enzyme	-	^246^
DNA	Nucleotide	45 nm–80 nm	^247^
Aspartate	Amino acid	30 nm	^248^
Phospholipid	Lipids	05 nm	^249^

### Bioreactors for green synthesis of gold nanoparticles

Phytomining is the approach through which plants can reduce metal ions both on their surface and in various organs and tissues remote from the ion penetration site. The metals like copper, gold, silver, platinum, iron, and many others accumulated by the plants can be recovered after harvesting methods. For example, *Brassica juncea* and *Medicago sativa*, both the plant accumulate 50 nm silver nanoparticles (13.6% of their weight) when grown on silver nitrate as a substrate whereas *M. sativa* accumulate 4 nm gold icosahedra,^250^ and Iris pseudacorus (yellow iris) accumulate 2 nm semi-spherical copper particles when grown on substrates containing salts of the respective metals. Few approaches have been demonstrated in which different varieties of plant extracts have been used in combination with different varieties of acids and salts of metals.^170,251^

## Factors affecting the formation of metal nanoparticles in plants

**Figure 4:**
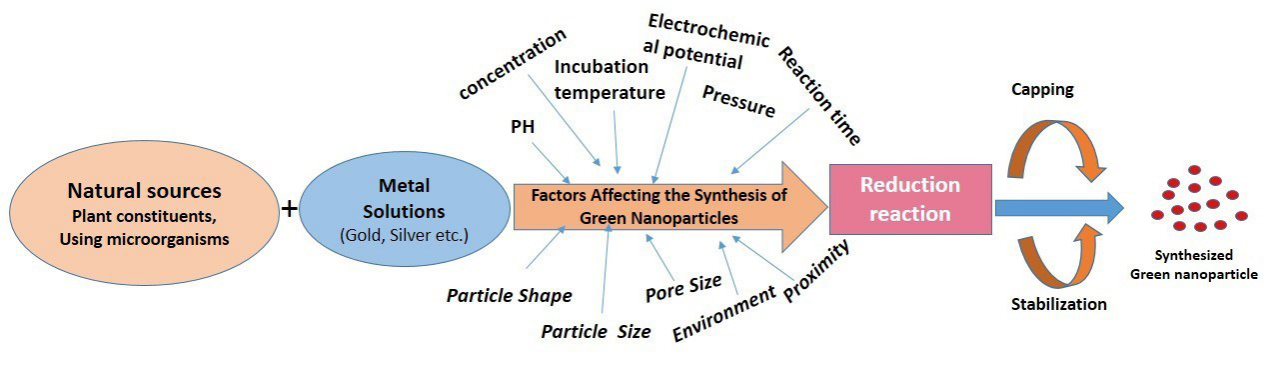
Factors affecting the formation of metal nanoparticles in plants.

Various limitations of nanoparticle synthesis by phytoconstituents are observed and it needed to be resolved carefully before industrial manufacture. The prime limitation is the intricacy in the identification of the phytoconstituents present in plants responsible for the NPs synthesis and therapeutic activity. The amount of reducing agent needs to be controlled because it hampers the reduction rate which results in the formation of large aggregated nanoparticles. Simultaneously the process parameter like thermal heating must be under controlled because during synthesis it can damage and denature various active molecules like sugars, and proteins resulting in the loss of activity. The reaction rate can be optimized by controlling the reduction reaction by varying the concentration of phytochemicals carefully. All the factors affecting the green synthesis of metal nanoparticles are presented in Figure 4.

To improve the efficacy, size and morphology of nanoparticles synthesized from biological sources by microorganisms several parameters need to be monitored like microorganism type, growth medium, growth stage (phase), synthesis conditions, reaction mixture pH, substrate concentrations, size, shape, incubation temperature and reaction time. The reduction process and stability of the biologically synthesized nanoparticles have a major concern and have to be controlled to improve the efficacy of the biologically synthesized nanoparticles. Major limitations in biologically synthesized nanoparticles are, the reduction process is quite slow and stable due to the decomposition of microorganisms over time.^111,157,252-254^

Nanoparticle aggregation is high at highly acidic pH over the reduction process and nucleation of reduced atoms. This may be related to the fact that a larger number of functional groups that bind and nucleate tetra-chloroauric acid ions become accessible at acidic pH.^115,255-257^ Efficiency and reaction rate of metal nanoparticle synthesis increases as an increase in the temperature. High temperatures required for crystal particle formation (nucleation rate is higher as increases the temperature). Interaction of phytochemicals with the nanoparticle surface may alter by elevated temperatures.^258-263^ Morphological diversity of the nanoparticles: triangles, hexagons, pentagons, cubes, spheres, ellipsoids, nanowires, and nanorods may occur due to the variation in concentration and composition of bioactive compounds present in plants.^252,264^

## Green synthesis of gold nanoparticles for tuberculosis

Apart from diversified biomedical applications, GNPs have been reported for antimicrobial activity against food and agriculture pathogens.^199^ Inherent property of antibacterial and antimicrobial^265^ activity of GNPs along with the entrapped plant extract, facilitate the early recovery from TB. The proposed mechanism for antibacterial activity of GNPs is that it increases gene expression in the redox process which leads to the death of bacteria and fungi. The nano size, surface area and photo thermic nature of GNPs directly influenced the antimicrobial activity.^266^ Another excepted mechanism is that intracellularly GNPs attached to the sulfur base present in cells in the form of thiol group in enzymes which leads the disturbance of respiratory chain suddenly by the generation of a large number of free radicals leading to death. On the contrary, the GNPs decrease ATP activities wherein they reduce the t RNA and ribosomal interaction. GNPs also block the transmembrane hydrogen efflux however lesser concentration of GNPs can inhibit bacterial growth about 250-fold. Due to the smaller size of GNPs then bacterial cells, they stick on the cell wall of pathogens and delay cell process, causing death. Some report shows a different mechanism when compared to other metal nanoparticles. GNPs due to the charge difference on the cell wall and nanoparticle surfaces it attracts bacterial DNA. On the other side, GNPs show the varied activity of gram-positive and gram-negative bacteria, which are classified based on the thick layer called peptidoglycan. Peptidoglycan generally consists of two joined amino sugars, N-acetylglucosamine and N-acetylmuramic acid (NAM), with a pentapeptide coming off the NAM forming an inflexible structure to diffuse the GNPs. Therefore, the peptidoglycan is very strong in gram-positive bacteria that penetrate GNPs across cell wall whereas gram-negative bacteria contain a thin layer which easily undergoes cell death. The anti-microbial activity also assisted by the concentration of capping agents and purification methods apart from the size and peptidoglycan thickness. In green synthesized GNPs the antimicrobial activity may be due to the synergistic effects of GNPS with plant extracts.^267^

The biophysical interactions between bacteria and nanoparticle occur through aggregation biosorption and cellular uptake that can damage the membrane and produce toxicity.^268^ The mechanism of antibacterial activity of the GNPs is attributed to the generation of reactive oxygen species that causes an increase of the oxidative stress of microbial cells and the release of intracellular lactate dehydrogenase enzyme into extracellular medium in form of vacuole formation as an indication of potent activity.^269-271^ Such effect was enhanced and exaggerated by photothermal degeneration in a combined approach, GNPs-laser, which causes quick loss of cell membrane integrity.^272^

GNPs have advantages over other metal nanoparticles because they are chemically inert, biocompatible nature and not producing cytotoxicity. Gold is used internally in humans for the last 50 years.^273^

Physical properties of the nanoparticle may differ from their corresponding parent materials by decreasing the size of nanoparticles and this relation offered many opportunities for many scientific breakthroughs. GNPs produced good antibacterial activity. It had been shown their best result when particles aggregation is not observed at high levels. GNPs with the same shape and size exhibited different inhibitory effects by changing surface modifications agents.^265^ It can also use in targeted molecular imaging in living subjects.^274^

**Figure 5:**
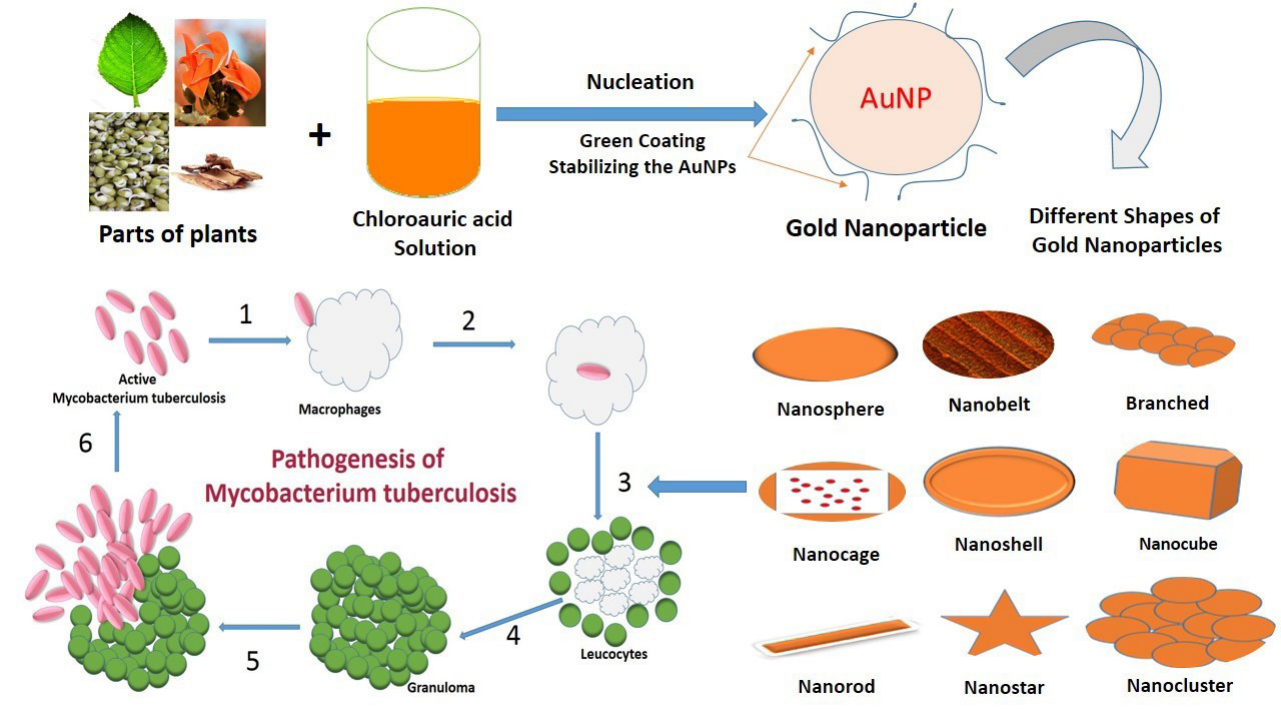
The summary of the review.

Recentely Gupta et al reported that the GNPs of ethanolic and hydroalcoholic exhibited anti-tubercular activity only at MIC 2.5 µg/mL and 20 µg/mL, respectively while ethanolic and hydroalcoholic extracts showed activity at much higher concentrations 50 µg/mL and 75 µg/mL, respectively.^275^ GNPs from *Nigella arvensis* (NA-GNPs) leaf extract were evaluated for antibacterial, antioxidant, cytotoxicity and catalytic activities and Chahardodli et al observed that NA-GNPs showed excellent cytotoxicity effects against H1299 and MCF-7 cancer cell lines with an IC50 value of 10 and 25 μg/mL, respectively and catalytic activity of NA-GNPs against methylene blue was 44%.^276^ Cheng et al synthesize GNPs using *Chenopodium formosanum* shell extract and concluded that GNPs possessed potent antibacterial activity against Escherichia coli and Staphylococcus aureus.^277^ Sunderam et al^278^ reported that green synthesized GNPs of *Anacardium occidentale* leaves extract, data presents good antibacterial effect against *Escherichia coli* and *Bacillus subtilis* and exhibited 74.47% viability on PBMC and 23.56% viability on MCF-7 cell lines at a maximum concentration of 100 µg/mL.^278^ Katas et al^279^ reported that the concentration of chitosan needed to synthesize antibacterial chitosan-GNPs with *Lignosus rhinocerotis* (LRE) was lower than those without LRE, suggesting that the addition of LRE as reducing agent resulted in higher antibacterial activity. Thus, chitosan as a stabilizing or capping agent and LRE as a reducing agent for the production of GNPs improved antibacterial activity of their resultant nanoparticles.^276-279^ Veena et al^280^ developed the green synthesis of *Vitex negundo* GNPs from leaf extracts and results exhibited strong antibacterial activity against gram-negative strains and moderate activity against gram-positive strains.^280^ The overview of the review is presented in Figure 5.

## Conclusion

The study of green synthesis of GNPs is a quickly evolving field in nanotechnology for TB. The present review summarises exhaustive literature for plants containing phytoconstituents having antitubercular activity along with the understanding of the synthesis of GNPs not only using plant extracts but biomolecules, microorganism, and various bioreactors. A detailed study is needed to give a lucid mechanism of biosynthesis of GNPs using biomolecules; microorganism present in different plant extracts which will be valuable to improve the properties of GNPs for TB treatment. With green chemical syntheses of these nanomaterials, researchers will able to conduct in-depth studies investigating biomedical applications without further biocompatibility preparations. In the coming years, the green chemistry procedure which utilizes plants their constituents, microorganisms, and biomolecules for nanoparticle preparation for TB has used as an alternative to conventional physicochemical methods since it is facile, rapid, cost-effective, and eco-friendly.

## Ethical Issues

Not applicable.

## Conflict of Interest

Authors declare no conflict of interest in this study.
